# Association Between Cardiac Surgeons’ Number of Years in Practice and Surgical Outcomes in New York Cardiac Centers

**DOI:** 10.1001/jamanetworkopen.2020.23671

**Published:** 2020-11-03

**Authors:** Gabe Weininger, Makoto Mori, Cornell Brooks, Michael Shang, Thais Faggion Vinholo, Yawei Zhang, Roland Assi, Arnar Geirsson, Prashanth Vallabhajosyula

**Affiliations:** 1 Division of Cardiac Surgery, Yale University School of Medicine, New Haven, Connecticut; 2 Center for Outcomes Research and Evaluation, Yale–New Haven Hospital, New Haven, Connecticut; 3 Section of Surgical Outcomes and Epidemiology, Yale School of Medicine, Yale School of Public Health, New Haven, Connecticut; 4 Yale Aortic Institute, Division of Cardiac Surgery, Yale School of Medicine, New Haven, Connecticut

## Abstract

**Importance:**

More than half of US cardiothoracic surgeons are older than 55 years, and the association between surgeon number of years in practice and surgical outcomes remains unclear.

**Objective:**

To assess the association between cardiac surgeons’ time in practice and operative outcomes for coronary artery bypass grafting (CABG) and valve surgery.

**Design, Setting, and Participants:**

Cross-sectional analysis performed of surgeon-level outcomes data from the 2014-2016 New York State Cardiac Data Reporting System across the 38 New York cardiac surgery centers. Years in practice were characterized as early career (<10 years) and late career (≥10 years). Participants were 120 cardiothoracic surgeons who performed CABG and 112 cardiothoracic surgeons who performed valve procedures between 2014 and 2016. Data were analyzed in April 2020. Surgeons who trained outside of the United States or had unclear training history were excluded.

**Main Outcomes and Measures:**

Risk-adjusted operative mortality rate (RAMR). Mortality was defined as all-cause death within 30 days of surgery or within the index hospitalization, whichever was longer. Risk adjustment was performed by a multivariable risk model developed by the New York State Department of Public Health. Restricted cubic spline curve identified the association between risk-adjusted mortality rate and surgeon number of years in practice. Linear regression models adjusted for surgeons’ annual case volumes.

**Results:**

A total of 112 CABG surgeons and 120 valve surgeons performed 39 436 CABG and 18 596 valve procedures between 2014 and 2016. The median number of surgeon years in practice was 20.0 (interquartile range [IQR], 12.0-28.5) years. The median surgeon annual case volume was 160.0 (IQR, 92.5-245.0) for CABG procedures and 104.0 (IQR, 43.0-210.0) for valve procedures. The median RAMR was 1.3% (IQR, 0.2%-2.2%) for CABG procedures and 3.1% (IQR, 1.7%-5.1%) for valve procedures. Surgeons with less than 10 years of practice had higher RAMR for valve procedures compared with surgeons with more than 10 years of practice (4.0 [IQR, 1.5-7.7] vs 2.9 [IQR, 1.7-4.7]; *P* = .20), but the finding was not statistically signficant. The RAMR for surgeons with less than 10 years of practice was similar compared with surgeons with more than 10 years of practice for CABG procedures (1.3 [IQR, 0.3-2.1] vs 1.3 [IQR, 0.0-2.2]; *P* = .73). A lower number of years in practice was significantly associated with higher RAMR for valve procedures (RAMR estimates for linear term: −1.144; 95% CI, −1.955 to −0.332; *P* = .006; quadratic term: 0.059; 95% CI, 0.015 to 1.102; *P* = .008; and cubic term: −0.001; 95% CI, −0.002 to 0.000; *P* = .01). This association was not observed for CABG.

**Conclusions and Relevance:**

In this cross-sectional study, compared with late-career cardiac surgeons, early-career cardiac surgeons were associated with worse risk-adjusted outcomes for valve operations but not for CABG. This finding suggests certain competence deficiency for valve surgery early after finishing training in cardiac surgery.

## Key Points

**Question:**

What is the association between cardiac surgeons’ years in practice and operative outcomes on coronary artery bypass grafting (CABG) and valve surgery?

**Findings:**

In this cross-sectional study of data from early-career (<10 years) and late-career (>10 years) cardiac surgeons practicing between 2014 and 2016 in New York, a lower number of years in practice for cardiac surgeons was significantly associated with a higher risk-adjusted mortality rate in valve procedures. The risk-adjusted mortality rate was similar across different numbers of years in practice for CABG procedures.

**Meaning:**

In this study, early-career status in cardiac surgeons was associated with worse surgical outcomes for valve operations, which suggests that additional complex valve surgery training in residency and mentorship guidance in early practice may be warranted.

## Teaser

This cross-sectional study uses surgeon-level outcomes data from the 2014-2016 New York State Cardiac Data Reporting System to assesses the association between surgeons’ number of years in practice and outcomes in cardiac surgery.

## Introduction

The association between surgeon experience and operative outcomes has been the subject of debate, with some studies suggesting worse outcomes with increasing surgeon age,^1,2,3^ while others report the opposite.^4,5^ A concave association, in which outcomes improve for the first number of years a surgeon is in practice, then plateau for a long period and worsen when surgeons approach retirement has also been reported.^3,6^ Many of these studies have used surgeon age, which may be confounded by various factors, including different training pathways and medical school starting age, as a surrogate for surgeon experience.^4,7,8,9^ A few studies have used years in practice, which measures surgeon experience more precisely.^3,5^

Most findings on this topic are based on investigations of general surgery cases. The association between surgeons’ number of years in practice and outcomes remains unclear in cardiac surgery, which has high-risk operations that may warrant examination. A few studies in cardiac surgery have examined coronary artery bypass grafting (CABG) outcome variation with conflicting results.^1,4,7^ However, to our knowledge, differences in the surgeon experience–outcome association for CABG and valve procedures have not yet been studied. Valve surgery carries a higher risk profile than CABG and is more often performed by more experienced surgeons.^9,10^ With more than half of the US cardiothoracic surgeons older than 55 years and nearing retirement,^11^ understanding this association is timely.

## Methods

We obtained surgeon-level outcomes and case volume data from the publicly available 2014-2016 New York State Cardiac Data Reporting System.^10^ Reported outcomes were observed mortality rate, expected mortality rate, and risk-adjusted mortality rate (RAMR) for isolated CABG and isolated valve or concomitant valve/CABG operations. All valve operations, excluding transcatheter procedures, were grouped into a single valve category in the New York State data. Both the expected mortality rate and RAMR were calculated from a multivariable risk model developed by the New York State Department of Public Health, accounting for patient demographic characteristics and comorbidities.^10^ Mortality was defined as all-cause death within 30 days of surgery or within the index hospitalization, whichever was longer.

All surgeon-level data were collected from 2014-2016 New York State outcomes data, the latest New York State surgeon-level outcomes report. Data were analyzed in April 2020. This study followed the Strengthening the Reporting of Observational Studies in Epidemiology (STROBE) reporting guideline for cohort studies. Because the data were publicly available, the Yale institutional review board waived approval and the need for patient consent.

**Figure 1.**
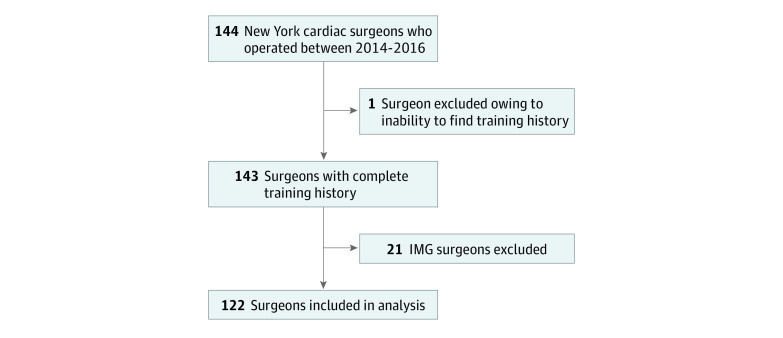
Inclusion Criteria for Surgeons Of the 122 total surgeons, 112 were included in coronary artery bypass graft (CABG) model and 120 were included in the valve model. The two groups overlap because most surgeons perform CABG and valve surgery. IMG indicates international medical graduates.

To determine surgeons’ number of years in practice, we obtained each surgeon’s medical school graduation year as well as residency and fellowship completion years from the Cardiothoracic Surgery Network website.^12^ Each surgeon’s final year of schooling was subtracted from 2016 (when the latest New York data were published) to determine number of years in practice. For surgeons whose training history was not listed on the Cardiothoracic Surgery Network website, we searched other online resources, such as the website of the surgeon’s current hospital and the Healthgrades website.^13^ We excluded international medical graduates because international medical graduates may have practiced as surgeons overseas, which may have obscured the actual years in practice. For surgeons practicing at multiple hospitals, we combined surgeon-level outcomes at those hospitals. Figure 1 depicts the inclusion criteria for surgeons.

Surgeon years in practice were categorized as early career (<10 years) and late career (≥10 years). The 10-year threshold was determined by the inflection point that occurred in the smoothed plot of risk-adjusted mortality and the number of years in practice.

### Statistical Analysis

Statistical analysis was performed using surgeon-level outcome data, surgeon case volumes, and surgeon years in practice. Continuous variables were summarized by median (interquartile range [IQR]) and categorical variables by percentages. Wilcoxon rank sum test and χ^2^ test were used to compare early-career with late-career surgeon status. We examined the association between surgeon-level RAMR and surgeon years of practice via linear regression models for CABG and valve procedures, adjusting for annual surgeon volume for respective case types. Years in practice was treated as a continuous variable and was modeled as a linear term for the CABG model and cubic term for the valve model to account for the nonlinear association with outcomes in valve operations. Although cumulative case volume in a surgeon’s career may mediate the association between experience and surgical outcomes, information on the cumulative case volume was not available in this cross-sectional data set. Therefore, such an association was not evaluated. All analyses were 2-tailed with statistical significance set at *P* < .05. Smoothed cubic spline associating mortality and the number of years was estimated using vcov package in Python 3.6 (Python Software Foundation). Analyses were performed using SAS, version 9.4 (SAS Institute Inc).

## Results

**Figure 2.**
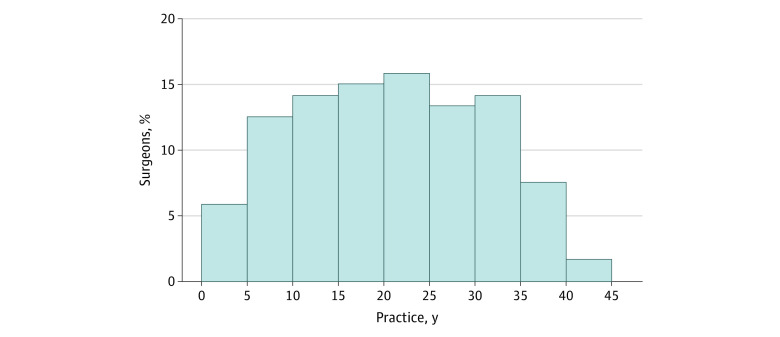
Distribution of Surgeons’ Years in Practice

A total of 112 CABG surgeons and 120 valve surgeons performed 39 436 CABG and 18 596 valve operations, respectively. The median number of years in practice was 20.0 (IQR, 12.0-28.5) years (Table 1 and Figure 2). The median observed mortality rate for CABG was 1.3 (IQR, 0.6-2.16) and the expected mortality rate for CABG was 1.44 (IQR, 1.22-1.65). The median surgeon annual case volume was 160.0 for CABG (IQR, 92.5-245.0) and 104.0 (IQR, 43.0-210.0) for valve procedures. The median RAMR was 1.3% (IQR, 0.2%-2.2%) for CABG and 3.1% (IQR, 1.7%-5.1%) for valve procedures (Table 1).

**Table 1.** Surgeon-Level Characteristics^a^

Variable	Median (IQR)			*P* value
	Early career^b^	Late career^c^	Total	
CABG				
Surgeons, No.	26	86	112	
Volume	127.5 (69.0-179.0)	179.5 (97.0-257.0)	160.0 (92.5-245.0)	.03
RAMR, %	1.3 (0.3-2.1)	1.3 (0.0-2.2)	1.3 (0.2-2.2)	.73
Valve				
Surgeons, No.	28	92	120	
Volume	52.0 (25.0-99.5)	127.5 (70.0-253.0)	104.0 (43.0-210.0)	<.001
RAMR, %	4.0 (1.5-7.7)	2.9 (1.7-4.7)	3.1 (1.7-5.1)	.20
Years in practice	7.5 (4.0-8.0)	24.5 (18.0-30.0)	20.0 (12.0-28.5)	<.001

Abbreviations: CABG, coronary artery bypass graft; IQR, interquartile range; RAMR, risk-adjusted mortality rate.

^a^ The surgeon characteristics are for all New York CABG and valve procedures between 2014 and 2016.

^b^ Early career is defined as less than 10 years in practice.

^c^ Late career is defined as 10 or more years in practice.

**Figure 3.**
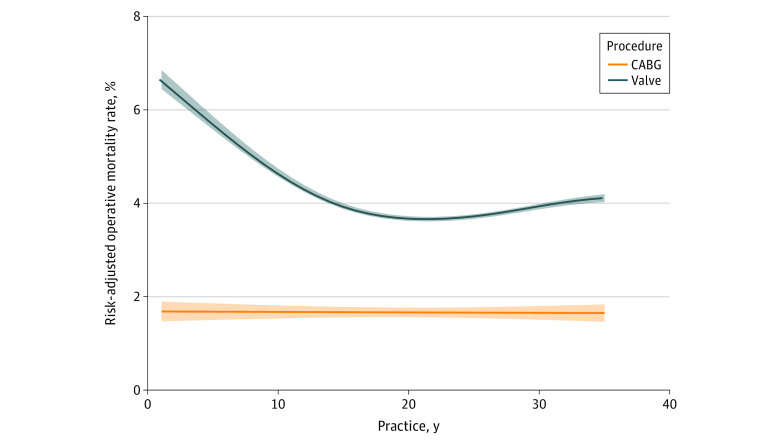
Association Between Number of Years in Practice and Risk-Adjusted Mortality Rate for Coronary Artery Bypass Graft (CABG) and Valve Procedures Restricted cubic spline fit of risk-adjusted mortality rate by years in practice for valve and CABG procedures. The bands represent 95% CIs.

Early-career status in surgeons had higher RAMR compared with late-career status in surgeons for valve procedures (4.0 [IQR, 1.5-7.7] vs 2.9 [IQR, 1.7-4.7]; *P* = .20), but the finding was not statistically significant. The RAMR was similar across the number of years in practice for CABG (1.3 [IQR, 0.3-2.1] vs 1.3 [IQR, 0.0-2.2; *P* = .73) (Figure 3). The valve model adjusting for case volume showed that lower number of years in practice was associated with higher RAMR in valve cases (RAMR estimates for linear term: −1.144; 95% CI, −1.955 to −0.332; *P* = .006; quadratic term: 0.059; 95% CI, 0.015 to 1.102; *P* = .008; and cubic term: −0.001; 95% CI, −0.002 to 0.000; *P* = .01). Higher case volume was associated with lower RAMR (−0.300 per 50-case increase; 95% CI, −0.531 to −0.070, *P* = .01). This association was not observed for CABG (Table 2).

**Table 2.** Regression Model for Surgeon RAMR^a^

Variable	RAMR estimate, % (95% CI)	*P* value
**Valve model (120 surgeons)**		
Years in practice^b^		
Linear term	−1.144 (−1.955 to −0.332)	.006
Quadratic term	0.059 (0.015 to 0.102)	.008
Cubic term	−0.001 (−0.002 to 0.000)	.01
Valve case volume (per 50-case increase)	−0.300 (−0.531 to −0.070)	.01
**CABG model (112 surgeons)**		
Years in practice^b^	−0.0442 (−0.145 to 0.056)	.38
CABG case volume (per 50-case increase)	0.0047 (−0.402 to 0.411)	.98

Abbreviations: CABG, coronary artery bypass graft; RAMR, risk-adjusted mortality rate.

^a^ The model for valve operations estimated a 0.3% decrease in RAMR per every 50-case increase in surgeon volume for valve operations.

^b^ Surgeons’ number of years in practice was modeled as a cubic term.

## Discussion

In this cross-sectional study using a large statewide data set, fewer years in practice was associated with worse risk-adjusted outcomes in valve surgery within the first 10 years in practice, but not for CABG. This finding was consistent after adjusting for case volume. In addition, extremely long years in practice was not associated with changes in outcome.

These findings are notable for several reasons. First, we were able to investigate the outcomes of valve operations in addition to CABG and show that these operations differ in their outcome association with surgeon experience, which, to our knowledge, has not been shown before. This difference is especially notable because valve surgeries have approximately twice the mortality of CABG^10^ and are more often performed by surgeons with a greater number of years in practice.^9^ Worse valve surgery outcomes for early-career surgeons may indicate the need for exposure to complex valve operations during training and appropriate supervision on patient selection and referrals during early years of clinical practice. In contrast, the lack of association between CABG outcome and surgeon years in practice suggests that current training models adequately prepare early-career surgeons to perform CABG.

Several options may address this potential gap in valve expertise. One option may be regionalization of complex valve operations, especially mitral valve. This approach may offer standardized training and mentoring structures within select centers while surgeons are in the early stage of their careers. Another option may be broader adoption of additional subspecialty fellowships after the general cardiothoracic surgery training, in which surgeons intending to specialize in valve operations would be encouraged to pursue additional training. Current cardiac surgery residency and fellowship programs may need to increase trainee exposure to complex valve operations. In addition, late-career surgeons who receive many valve referrals could be encouraged to involve and share cases with early-career surgeons who receive fewer valve referrals and may learn from increased exposure.

Our results are consistent with studies reporting low case volume being associated with worse outcomes in valve surgery.^14,15^ However, our finding that CABG outcomes are not significantly associated with surgeon experience differs from previous studies that have reported mixed results, some of which show worse outcomes for older surgeons and some of which show improved outcomes.^5,7,9^

Two studies that found worse CABG outcomes to be associated with older surgeons used outcome data from 1989 to 1992 and from 1998 to 1999,^1,9^ which may not reflect contemporary practice patterns and training paradigms. Tsugawa and colleagues^7^ used Medicare data from 2011 to 2014 and reported that older surgeon status was associated with obtaining better CABG outcomes. Differences between this study and ours may owe to the Tsugawa and colleagues^7^ data set including only nonelective procedures for Medicare patients (age ≥65 years) in contrast to our data, which included elective and nonelective procedures performed on patients of all ages.

### Limitations

This study has limitations. Our data set was a mandatory statewide outcome reporting system, which minimized potential selection bias or a narrow scope of payer-specific databases such as Medicare data. However, the limitations of this data set include the use of single-state data, which has a limited sample size of surgeons and may not represent states with practice and referral patterns different from New York. In addition, the valve surgery outcome data are reported as a combination of mitral, aortic, and tricuspid valve operations, and risk-adjusted outcomes for each valve operation type could not be evaluated. Valve and combined valve/CABG cases were also grouped into a single category such that these 2 case types could not be evaluated individually. The sample size of surgeons with less than 10 years in practice was 28 for the valve model, which could be subject to variability, although statistical tests show significant results. Further, a variety of factors may affect surgeon-level outcomes that we were not able to measure, such as cases with cosurgeons, number of revision operations, quality of residency training, and size of hospital program.

## Conclusions

In this cross-sectional study, early-career status in surgeons with fewer than 10 years in practice have worse risk-adjusted mortality for valve operations but not for CABG surgery. These findings suggest that additional valve surgery training in residency or close mentorship for early-career surgeons on valve operations may be warranted.
